# Development of the Canadian food intake screener for adolescents based on Canada’s Food Guide 2019 healthy eating recommendations

**DOI:** 10.1186/s12966-025-01837-1

**Published:** 2025-10-21

**Authors:** Claire Tugault-Lafleur, Virginie Desgreniers, Geneviève Bessette, Rita Al Kazzi, Raphaëlle Jacob, Kimberley Hernandez, Sylvie St-Pierre, Jess Haines

**Affiliations:** 1 School of Nutrition, Faculty of Health Sciences, University of Ottawa, 200 Lees Ave, Ottawa, ON K1S 5S9 Canada; 2 Department of Family Relations and Applied Nutrition, University of Guelph, Guelph, ON Canada; 3 Health Canada, Government of Canada, Ottawa, ON Canada

**Keywords:** Questionnaire development, Children, Adolescent, Cognitive interviews, Content validity, Canada’s food guide

## Abstract

**Background:**

Assessing adolescents’ dietary intakes in relation to Canada’s Food Guide 2019 (CFG-2019) recommendations on healthy food choices is a critical component to public health surveillance efforts. The study aimed to develop a brief self-administered screener to assess food intake based on CFG-2019 food choices recommendations among English- and French-speaking adolescents aged 10–17 years living in Canada.

**Methods:**

The development and assessment of the content validity of the tool was undertaken in collaboration with Health Canada advisors and informed by external content experts, including nutrition researchers and practitioners. Following a rapid review of screeners used among children aged 6–17 years, an initial draft was developed, and content validity was assessed by an expert panel with expertise in public health nutrition and questionnaire validation (English *n* = 13, French *n* = 7). Two rounds of cognitive interviews were then conducted with adolescents (English *n* = 15, French *n* = 14) to assess comprehension and further refine the screener items. Cognitive testing using a direct probing approach was conducted iteratively in two phases to assess understanding of questions and incorporate feedback from adolescents to improve the clarity and wording of the items at each phase.

**Results:**

Following the expert panel and iterative discussions with Health Canada advisors, four items were removed from the initial 14-item screener as these items were deemed not sufficiently reflective of the CFG recommendations and one item asking about water intake was tested. Cognitive testing revealed that the items were well understood overall, and feedback at each interview round enabled additional refinements to improve comprehension. The resulting screener includes 10 items designed to rapidly assess food intake based on CFG-2019 recommendations on healthy food choices for adolescents aged 10 to 17 years.

**Conclusions:**

The Canadian Food Intake Screener for Adolescents/*Questionnaire court canadien sur les apports alimentaires des adolescents* is designed to rapidly assess dietary intake over the past week among children aged 10 to 17 years. Before it can be used for research and population-level nutrition surveillance, further research is needed to develop a scoring system and evaluate the screener’s construct validity and reliability.

**Supplementary Information:**

The online version contains supplementary material available at 10.1186/s12966-025-01837-1.

## Introduction

Assessing how well dietary intakes of adolescents align with national dietary guidelines is a critical component to food and nutrition surveillance efforts. Analyses from nation-wide dietary surveys have shown that despite modest improvements [1, 2], Canadian adolescents have room to improve their dietary quality [3–5].

Assessing what children eat requires instruments that are valid, reliable and adapted to this life stage. As children transition from childhood into adolescence, there is a rapid increase in the cognitive ability of children to self-report food intake [6]. Adolescence is a key developmental period in which issues such as lower engagement, and concerns about body image may affect dietary reporting [6, 7]. Special considerations should thus be taken when choosing or developing a dietary assessment tool targeting this age group.

Brief dietary screeners enable rapid assessment of food intake in settings where comprehensive methods, such as 24-hour recalls, are impractical [8, 9]. In contrast to more time-consuming and resource-intensive methods such as 24-hour dietary recalls—which aim to capture diet comprehensively on a given day— dietary screeners, though less precise, are designed to assess dietary intakes over a more prolonged period, such as over the past month or week [8, 9].

Recently, the Canadian Food Intake Screener/*Questionnaire court canadien sur les apports alimentaires* was developed to assess how well adults’ dietary intakes align with healthy food choice recommendations outlined in Canada’s Food Guide 2019 (CFG-2019) [10]. The healthy food choice recommendations contain advice on the types of foods and beverages to eat and to limit each day [11]. Canadians are encouraged to consume vegetables and fruits, protein foods and whole grains, and to limit highly processed foods such as sugary drinks, baked goods, fast foods and processed meats.

The Canadian Food Intake screener was designed for use in research and public health surveillance contexts in which comprehensive dietary assessment methods are not always feasible. Due to capacity, time and resource constraints, the targeted populations for this screener could not include children and adolescents. Therefore, the purpose of this study was to develop a self-administered screener designed to assess the food intake of Canadian children aged 10 to 17 years based on CFG-2019 healthy food choice recommendations. This paper describes the development process and the content validity of the screener. A separate brief questionnaire, the Canadian Eating Practice Screener for Adolescents, was developed to assess children’s alignment with CFG-2019 recommendations on eating practices and is described in the accompanying paper (Jacob et al., under review).

## Methods

**Fig. 1:**
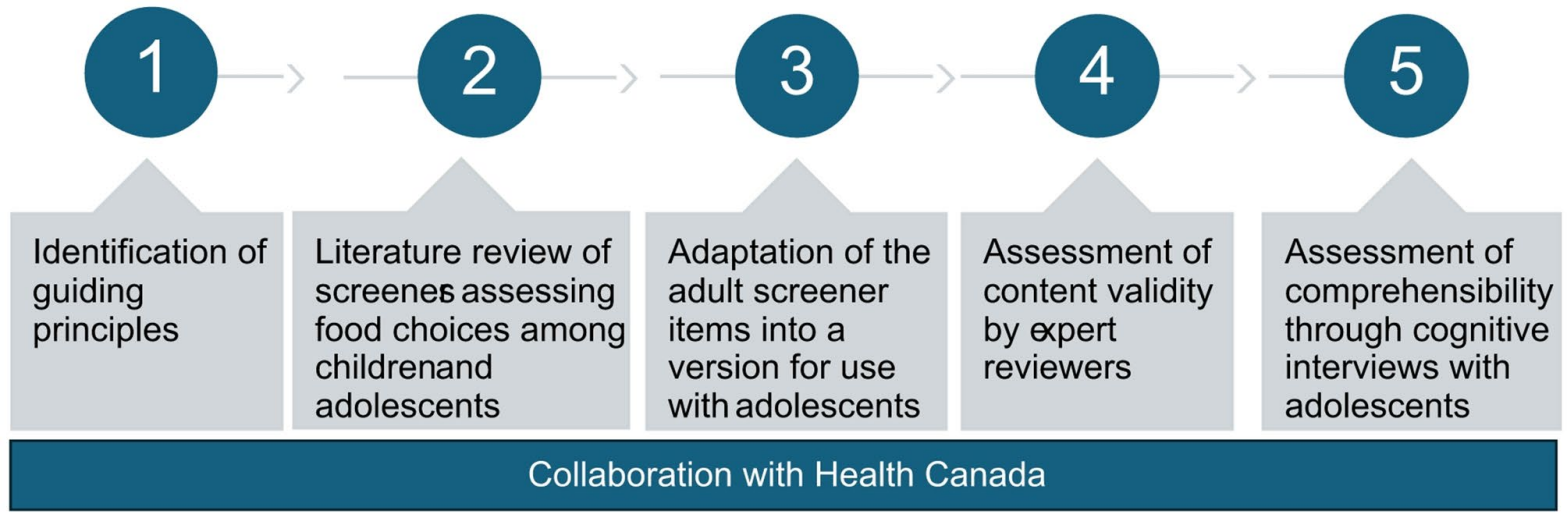
Process for development of a brief measure designed to assess alignment of adolescents’ dietary intakes to Canada’s Food Guide 2019 recommendations on healthy food choices

The development and assessment of the content validity of the tool was undertaken in collaboration with Health Canada advisors and informed by external content experts, including nutrition researchers and practitioners. The 5-step process in the development of the screener is illustrated in Fig. 1. The first step involved the establishment of guiding principles, followed by a literature review of brief self-administered food intake screeners used with children aged 6–17 years. We then developed a first version of the screener by modifying the adult version of the screener [10, 12]. Steps four and five involved the assessment of content validity through an expert panel and cognitive interviews with children aged 10–17 years, respectively.

### Defining guiding principles

A set of guiding principles, defined a priori in collaboration with Health Canada advisors, was developed to guide the development of the screener. These guiding principles were as follows: (1) the screener should be simple to use (administer and complete), (2) be brief (i.e. take no longer than 5 min to complete), (3) assess food intake relevant to CFG-2019, (4) consider the cognitive demand, numeracy and literacy of children aged 10 to 17 years, (5) be easily understood by adolescents as determined through cognitive interviewing, and (6) be sensitive to needs and life stages of adolescents (i.e. ensure use of sensitive and relevant language, such as gender identity and weight bias).

### Rapid review of food intake screeners used among children aged 6–17 years

A rapid review was conducted by Health Canada to summarize existing diet screeners to rapidly assess food intakes of children aged 6–17 years. The purpose of the review was to provide insights regarding key characteristics such as screener length, target age range, recall period, and consideration for validity. The search was performed from May to August 2023, and a total of 31 screeners were reviewed [13]. Screeners reviewed contained a median number of 16 items. The review confirmed that children begin to self-report their own intake using a frequency-based instrument at around 10 years of age [14–17]. From the 31 screeners reviewed, 40% assessed intake during the previous week [13], which confirmed the need to adjust the recall period from the previous month to the previous week. Many of the screeners reviewed were multifactorial (meaning that children were asked to report on several dietary components within a screener) [14, 17–20].

### Adaptation of the Canadian food intake screener

An initial version of the screener was developed by modifying the items, instructions and response options from the Canadian Food Intake screener for adults [10, 12]. As in the adult food intake screener, each item was mapped to the Canadian healthy eating recommendations (e.g., “Eat plenty of vegetables and fruit”, “Eat whole grain foods”). The 14 items in the initial draft for adolescents were similar in content to the adult screener, except for the following: (1) the recall period was changed to assess intake from the previous week instead of the previous month; (2) two items from the adult screener were excluded as they were deemed not directly relevant for adolescents based on the CFG-2019 healthy food choice recommendations for children and adolescents [21]. Health Canada then translated the initial screener draft into French. The translated document was subsequently reviewed by bilingual members of the research team prior to the first round of cognitive testing.

### Assessment of content validity

Content validity is the degree to which a questionnaire adequately reflects the concepts being measured within the tool. Measuring content validity includes assessing whether the questionnaire captures the construct(s) to be measured in terms of relevance, comprehensiveness and comprehensibility [22–25]. Content validity testing with experts investigated relevance, comprehensiveness and comprehensibility of the tool while cognitive interviews with adolescents later focused on comprehension by the target population.

Twenty panelists with expertise in childhood and adolescent nutrition, public health, and questionnaire validation (English *n* = 13, French *n* = 7) were identified by members of the research team based on existing contacts and invited by email to participate (Additional File 1). All experts agreed to participate and were sent a form within a Microsoft Excel (Microsoft Corporation, Redmond, WA) spreadsheet. The form asked experts to comment on whether each item adequately reflected a healthy eating recommendation within the CFG-2019 (e.g., “eat plenty of vegetables and fruit”). Experts were also asked whether each item was clear and easy to understand, with yes/no response options for each item. Finally, experts were invited to provide additional suggestions for improvements to each item and comment on whether important concepts of the recommendations were missing.

The collated feedback from experts was shared and discussed among members of the research team and Health Canada advisors to achieve consensus on whether to remove or modify any item that was poorly rated by the expert panel. A threshold of more than 75% was deemed sufficient agreement to conclude that an item was relevant and clear [26]. However, we considered all written feedback from experts for each item (regardless of the agreement rating). Following the meeting, the screener was reviewed by Health Canada staff to check for policy relevance as well as to improve readability and further adapt language to a lower grade level (e.g., break down long sentences, use colloquial terms).

### Assessment of comprehensibility via cognitive interviews

Cognitive testing is a qualitative method that allows one to determine whether a question is understood consistently in the way researchers intend with the end users—in this case, English- and French-speaking adolescents [27–30]. Cognitive interviewing also allows researchers to identify and correct potential sources of errors, which then increases the content validity of the questions [30, 31]. Tourangeau’s four-stages model describes these processes in more detail, which include comprehension, retrieval of information, judgement or estimation, and selection of a response to the question [32]. These stages of cognitive processing can be explored using probing (direct questions) or ‘Think Aloud’ methods in which participants are asked to explore ways in which they obtained their answers [30]. In the context of this study, a direct verbal probing method was used with adolescents to reduce participant burden.

#### Participants

Recommendations for sample sizes when conducting cognitive testing are variable, but previous research has recommended a range from 10 to 30 participants, with five to 15 participants per round for two to three rounds [29–31]. In the current study, we conducted interviews for two rounds (with 6 to 8 participants per round in each language) to allow for iterative improvement [31]. We did not preset the number of rounds, but we expected that two to three rounds would be sufficient to ensure adequate content validity.

Participants were recruited through community and parent-focused organizations and social media groups on Facebook, word of mouth, as well as direct and mass email (e.g., the University of Ottawa’s employee listserv) from March to April 2024. Eligible individuals were aged 10–17 years, lived in Canada, and were able to read the screener and complete a 30–45-minute interview using online teleconferencing software [Microsoft Teams (Microsoft Corporation, Redmond, WA)] in English or French. A quota sampling approach was used for balanced gender for each language group. We aimed to have balanced age groups so that each interview round would have both younger (10–13 years of age) and older (14–17 years of age) adolescents. Finally, we also aimed for diversity in terms of racial groups within our sample of English- and French- speaking adolescents.

Potential participants were invited to write an email to the research team who then assessed eligibility by confirming the age and gender with the parent or the adolescent themselves if they had reached out directly to the research team. Eligible individuals were then sent a consent form (intended for parents) and an assent form (intended for adolescents) to review. Once the consent form was received, the research team sent out an email invitation to participate in an online interview. The assent form was reviewed at the beginning of the interview, at which time the participant was asked to provide verbal informed consent. Participants who completed an interview received a $25 CAD Amazon gift card in appreciation of their time. Ethics approval was obtained at the University of Ottawa (H-11-23-9832), the University of Guelph (certificate number 21-02-010) and Health Canada’s Research Ethics Board (certificate number REB 2023–036 H).

#### Data collection

Cognitive interviews were conducted by a team of bilingual researchers at the university of Ottawa (CTL, DV, RAK, GB) from March to April 2024. Two research assistants were present during each interview, assuming the roles of a lead interviewer and a note-taker, to capture details of participants’ responses. Interviews were video- recorded.

The interview process used a standard approach – first asking adolescents to provide information on their age, gender, ethnicity, and whether they were able to respond to survey questions in the language in which the interview was conducted (English or French). The lead interviewer used the shared ‘share screen’ function in MS Teams and reviewed the assent form with the participant, explaining how the interview would be conducted, and obtained their verbal assent to participate in the study and to video record the interview. Before starting the recording, a practice question was conducted with the interviewee. The lead interviewer then displayed the screener instructions first, which was followed by each item with its response scale. Each interviewee first read the question (out loud or in their head) and provided a verbal answer to that question. The interviewer then used verbal probes to verify the comprehension, retrieval of information, judgement or estimation, and response selection for each item [32]. For example, to assess comprehension, participants were asked, “In your own words, what is this question asking?” and “Was there anything confusing about this question?”. If a word was suspected to be misunderstood by participants, interviewers asked “what does the word [X- will fill in the word or phrase relevant to each question] mean to you?” or “In this question, we used the phrase [X]. What does this phrase mean to you?”. Retrieval of information was assessed by asking participants, “Was it easy or hard to decide what your answer should be? What made it [easy/hard]?”. Finally, judgement and selection of the response was assessed by asking participants, “Are the response choices clear? How would you make the response choices easier to understand?”. Each interview lasted approximately 30 min.

Following the interview, the interviewer and note-taker completed a debriefing form to capture detailed notes on the interview process. By the end of the second round in each language, diminishing returns [31] were noted, in that few new problems were being identified, and recruitment and data collection were concluded.

#### Data analysis

Using the same approach as for the development of the Canadian Food Intake Screener for adults [10], the interview notes and recordings were informally coded. Following each round of interviews in French and English, the research team met to identify issues that required changes to ensure that each item would be understood as intended [29, 33]. The research team also met with Health Canada advisors after each round to discuss these issues and suggested modifications to the screener before the next round of interviews. When modifications were made in one language, the corresponding changes were made in the other language to ensure translational equivalency [34, 35]. For reporting purposes, the issues identified were subsequently grouped into problem categories or themes related to cognitive processes [29, 33, 36].

## Results

### Findings from the expert panel

Among English panel experts, one item (out of the 14) did not meet the minimum threshold for being sufficiently reflective of the recommendations, and one did not meet the minimum threshold for being clear/easy-to-read. Within the French panel, all items met the threshold for being relevant, but three items ranked poorly for being clear/easy to read. Finally, the experts’ feedback provided suggestions on simplifying some of the vocabulary and using more colloquial terms to ensure that the item would be understood by adolescents with lower literacy levels.

The research team presented these findings to a Health Canada advisor meeting. Following the discussion, four items querying about specific food subgroups (white milk, chocolate/flavored milk, yogurt/cheese/kefir and refined grains) were removed as they were deemed not sufficiently reflective of the healthy eating recommendations. Some of the items asking about a specific food were added in to another item. For example, the item asking about white milk and unsweetened plant-based beverages was added into an item asking about protein foods (since the CFG-2019 does not make explicit recommendations regarding milk intake). One item inquiring about the frequency of water consumption was developed from a previous beverage screener developed for adolescents [37] to reflect the CFG recommendation of “making water your beverage of choice”. A total of 11 items were tested during the first round of cognitive interviews.

### Findings from cognitive interviews with adolescents

A total of 29 interviews (15 in English and 14 in French) were conducted in March and April 2024 (Table 1). In total, 15 participants identified as girls,14 as boys, and none identified as non-binary. A larger proportion of participants were younger (10–13 years) compared to older (14–17 years) adolescents. We identified several cognitive issues with the screener items during interviews, which can be summarized in the following themes: lack of clarity about what to include or exclude within food groupings, keyword confusion, and readability. These issues are presented in Table 2 and discussed in more detail below.

**Table 1:** Characteristics of participants (*n* = 29) in cognitive interviews in english and French to evaluate the content and comprehensibility of the Canadian food intake screener for adolescents

	English *n* = 15	French *n* = 14	Total sample *n* = 29
Age group			
10–13 years	9	12	21
14–17 years	6	2	8
Gender			
Girls	9	6	15
Boys	6	8	14
Race/Cultural group			
White	11	14	25
Asian	1	0	1
Latino	2	0	2
Unknown to participants	1	0	1

**Table 2:** Examples of issues identified in cognitive interviews with English- and French-speaking participants when evaluating the comprehension of the Canadian food intake screener for adolescents (*n* = 29 participants)

Example	Problem category	Focus of question	Cognitive issue	Modification
1	Lack of clarity about what to include or exclude/food groupings	Fruits	The initial version of the screener asked, “How often did you eat fruit in the past week? Do not include fruit juices or drinks.” (in French, “Combien de fois as-tu mangé des fruits durant la dernière semaine? N’inclus pas les jus de fruits et les boissons aux fruits.”). Several participants said they only included fresh (and not frozen or canned fruits) in their responses.	The item was updated to be more specific about what type of fruits to include (fresh, frozen, canned and dried).
2	Lack of clarity about what food to include or exclude/food groupings	Vegetables	The initial version of the screener asked, “How often did you eat vegetable in the past week? Do not include deep-fried vegetables like French fries, or vegetable juices and drinks.” (In French, “Combien de fois as-tu mangé des légumes durant la dernière semaine? N’inclus pas les légumes frits comme les frites ni les jus ou cocktails de légumes.” In the first round of cognitive interviewing, probing questions were used to ask whether youth were including not only fresh vegetables but also frozen or canned vegetables in their responses. Some youth did while others did not. Youth preferred to have the question be specific about what vegetable to include. Probing questions were asked to see whether youth were including potatoes in their answers, and some did while others did not.	The item was updated to be more specific about what type of vegetables to include (fresh, frozen, canned and dried).
3	Lack of clarity about what to include or exclude/food groupings	Foods from restaurants and fast-food restaurants	The initial version of the screener asked, “How often did you eat foods from restaurants or fast-food restaurants in the past week? Examples of these foods include hamburgers, French fries, fried chicken, or tacos.” (In French, “Combien de fois as-tu mangé des aliments provenant de la restauration rapide ou de type ‘fast food’ durant la dernière semaine? Voici quelques exemples de ces aliments : hamburgers, frites, poulet frit, tacos.” French participants were not sure whether to include ready-to-eat foods and meals bought in grocery stores such as frozen pizzas or chicken nuggets in their response. Participants suggested adding more examples of commonly consumed fast foods such as pizza, poutine, as well as sweet foods such as ice cream.	A modification was made to the question to clarify that the question was asking about foods prepared outside the home. Additional examples were added to help with recall.
4	Lack of clarity about what to include or exclude/food grouping (leading to response errors)	Sugary drinks	The initial version of the screener asked, “How often did you have sugary drinks in the past week? Examples include: iced tea, fruit juice, fruit-flavoured drinks like fruit punch, soda or pop, sports drinks, energy drinks, hot chocolate, chocolate milk, specialty coffee and teas, flavoured waters with added sugars, sweetened plant-based beverages.” (In French, “Combien de fois as-tu bu des boissons sucrées durant la dernière semaine? Voici quelques exemples de ces boissons : thé glacé, jus de fruits, boissons aromatisées aux fruits, boissons gazeuses, boissons sportives, boissons énergisantes, chocolat chaud, lait au chocolat, café ou thé contenant du sucre ajouté, eau aromatisée sucrée, et boissons d’origine végétales sucrées.”) In the first round of cognitive interviewing, probing questions were used to ask whether participants were including diet sodas or pop in their answers. Responses were mixed (some did while others did not).	The item was modified by changing the term “sugary drinks” to “sweet drinks” to include all beverages that were either sweetened or tasted sweet such as beverages with artificial sweeteners. In the list of examples, the term “regular/diet” was added in front of soda or pop.
5	Keyword confusion	Processed meats, corned beef, packaged plant-based meats, charcuteries	The initial version of the screener asked, “How often did you eat processed meats in the past week? Examples of these foods include hot dogs, sausages, ham, corned beef, beef jerky, bacon, and other deli meats. Do not include canned fish or canned poultry or packaged plant-based meats.” (In French, “Combien de fois as-tu mangé des viandes transformées durant la dernière semaine? Voici quelques exemples : hot-dogs, saucisses, charcuteries (jambon, bacon, salami). N’inclus pas le poisson ou le poulet en conserve, ni les aliments protéinés d’origine végétale transformés. »). Although participants appreciated the list of examples to help understand what processed meats were, other keywords such as “corned beef”, “packaged plant-based meats” and “charcuteries” were not well understood.	The list of food items was updated to move deli meat earlier in the list with some examples provided within parentheses. The terms “corned beef” was replaced with “pastrami/smoked meat”. In French, the term “viandes froides” was added next to “charcuteries” to help youth understand what type of processed meats to include in their responses.
6	Keyword confusion	Protein foods that come from plants	The initial version of the screener asked, “How often did you eat protein foods that come from plants in the past week? Examples of these foods include: nuts and seeds, beans, peas and lentils, fortified soy beverages, tofu, soybeans and other soy products. Do not include green beans and packaged veggie burgers and plant-based meats.” (In French, “Combien de fois as-tu mangé des aliments protéinés d’origine végétales durant la dernière semaine? Voici quelques exemples : noix et graines, légumineuses, boissons de soya enrichies, tofu ou autres produits à base de soya. N’inclus pas les hamburgers végétariens transformés et les viandes d’origine végétale. ”) English-speaking participants felt the wording to be overly complicated and preferred simply saying “plant-based protein foods”.	The term “protein foods that come from plants” was changed in English to “plant-based protein foods”. No changes were made to the French screener since youth said they understood the term “aliments protéinés d’origine végétales”.
7	Keyword confusion	Peas, legumes	English-speaking participants found the term “peas” confusing when talking about dried pulses. Among French participants, the term “légumineuses” (in English, legumes) was not well understood.	In the list of food examples, “beans, peas and lentils” was changed to “beans, lentils, chickpeas” (in French, “fèves/haricots secs, lentilles, pois chiches”).
8	Keyword confusion	Whole grains	The original version of this item asked, “How often did you have whole wheat or whole grain foods in the past week? Examples include whole wheat or whole grain: breads, bagels, pasta, noodles, quinoa, oats, brown or wild rice, breakfast cereals. Do not include white/refined breads, bagels, pasta, noodles, rice, or refined breakfast cereals.” In French, “Combien de fois as-tu mangé des aliments à blé ou grains entiers? Inclue les pains, bagels, pâtes, nouilles et céréales à déjeuner à blé entier ou grains entiers, le quinoa, l’avoine, le riz brun et le riz sauvage. N’inclus pas les pains, bagels, pâtes et nouilles blanches, les céréales à déjeuner raffinées et le riz blanc.” Participants felt like the second part of the question (“Examples include…”) was not well formulated which led to some confusion. Participants felt like adding the terms “whole wheat or whole grain” in front of breads and pasta and noodles would help clarify which foods to include in their answer. Finally, participants suggested removing the last part around foods to not include (white breads) as they felt like the item was clear and the item was already long to read.	The item was simplified and asked, “How often did you have whole wheat or whole grain foods in the past week? Whole wheat or whole grain foods include: whole wheat or whole grain breads, whole grain pasta and noodles, quinoa, oats, brown or wild rice, whole grain breakfast cereals”. The item was simplified, and the list of exclusions was removed (“Do not include white/refined breads, …”) in both English and French versions of the item.
9	Readability	All items with a list of food examples	Participants liked the list of food examples to help with recall but noted that these took a long time to read.	The list of food examples was changed to a bullet list to enhance readability.

#### Lack of clarity about what foods to include or exclude in responses

Participants expressed some concerns over the lack of clarity about what foods to include or exclude in their responses for some of the items. That is, they felt that greater detail could be added to reduce the confusion or proposed more food examples to clarify what was meant with a particular food grouping (Table 2, examples 1–3). For example, for one question asking about the frequency of eating at restaurants and fast-food restaurants, three French-speaking participants in the first round of interviewing asked whether to include ready-to-heat fast foods in their answer (for example, ready-to-heat frozen pizzas or chicken nuggets bought at the grocery store but prepared at home) (Table 2, example 3**)**. A modification to the question in French was made to clarify that the question was focused on foods that came from fast food restaurants. For the item related to the consumption of sugary drinks (Table 2, example 4), some participants felt like the term “soda or pop” was ambiguous and they were not sure whether to include diet (sugar-free) soda or pop that still tasted sweet. These ambiguities were addressed by adding detail to existing questions or through formatting of the questions. In the latter example, the term “regular/diet” was added, and “sugary drinks” was replaced by “sweet drinks” to indicate that the question referred to any beverage that tasted sweet.

#### Keyword confusion

Some keywords in the initial version of the screener were unclear or confusing to participants (Table 2, examples 5 and 6). Clarity was improved by replacing some of the keywords (e.g., “corned beef” was replaced with “pastrami/smoked meat”) or removing a keyword that was not well understood by participants (e.g., “packaged plant-based meats”). Specific foods were added to the list of food examples to enhance clarity (“beans, peas and lentils” was replaced with “beans, chickpeas and lentils”) (Table 2, examples 6 and 7). For one item asking about whole grain intake, participants suggested adding the term “whole wheat or whole grain” in front of food examples such as bread, pasta and noodles to help clarify what whole grain foods were.

#### Readability

When encountering lengthy lists of examples (for most items), participants tended to miss details and consequently provided inaccurate responses (Table 2, example 9). These issues were mitigated by formatting changes (listing examples of foods using bullet points) and by re-wording and simplifying phrasing (e.g., reducing the number of examples).

### Final screener

The final version of the screener in both languages includes 10 questions. Five questions assess foods that should be consumed often according to CFG-2019 recommendations on healthy food choices (fruits, vegetables, protein foods, plant-based protein foods and whole-grain foods). The same response scale is consistent throughout all items, ranging from “Never” to “6 or more times per day” to decrease cognitive load for participants. Five items assess foods to limit (processed meat, fast food, sweet drinks, sugary snacks, salty snacks). Although the item asking about water intake was well understood by participants, it was removed from the final screener as a substantial ceiling effect was observed (most participants stated they drank “6 or more times per day”). The final version of the screener, in both English and French, can be found in Additional File 2.

## Discussion

Valid and reliable tools to measure food intake of adolescents are crucial to nutrition and surveillance efforts in Canada. However, this demographic is heterogenous, with a wide range of developmental capabilities and varying motivation to engage in dietary assessment [7]. The Canadian Food Intake Screener for Adolescents/Questionnaire court canadien sur les apports alimentaires des adolescents, available in English and French, rapidly assesses dietary intake during the past week and is informed by food choice recommendations from CFG-2019. The screener is intended for use in research with children aged 10 to 17 years and was developed through an iterative process that included continuous feedback from advisors, content validity testing via a panel of experts, and comprehensibility testing through two rounds of cognitive interviews in two languages simultaneously. Findings suggest that the screener is well understood in both French and English, and continual refinement helped improve the wording of the items, minimize cognitive load and improve comprehension.

Conducting an evidence summary on existing screeners was a critical step in exploring ways of adapting the adult food intake screener for our target population. For example, the review confirmed that children begin to self-report their own intake using a frequency-based instrument at around 10 years of age, but that a recall period of one month would need to be shortened to intake over the past week. The literature review step also confirmed that adolescents are able to report on a variety of food groups, as the majority of food intake screeners reviewed were multifactorial.

The expert assessment and ongoing input from advisors from Health Canada facilitated refining the initial items and prioritizing those that directly assessed the CFG-2019 recommendations on healthy food choices most relevant for adolescents. Advisor feedback helped ensure items were clearly phrased using accessible language and familiar food examples. Similarly, as in the development of the adult version of the food intake screener, an effort was made to balance trade-offs between being specific with longer food lists and minimizing cognitive load which could result in more reporting error [38].

Cognitive interviewing was a critical step in the development of the screener. Participants provided useful suggestions to improve the language structure, decrease keyword confusion (e.g., corned beef, luncheon meats) and incorporate their perspective to improve the readability of the items (e.g., by using bullet lists). Conducting the interviews in two languages also provided unexpected benefits in terms of rephrasing an item to make it easier to read and understand in both languages. Specifically, some suggestions provided by adolescents for rephrasing an item in one language also helped make the item easier to read and understand in the other language.

Comments from participants that arose during interviews were generally similar between language groups. Overall, both French- and English- speaking participants reported the items as being clear and easy to understand. However, both language groups reported difficulties understanding specific terms such as processed meats (“viandes transformées”). Both groups appreciated the list of food examples to help them with recall (e.g., protein foods, whole grains) and remember what foods to include in their answers. Nonetheless, we found differences across groups in the perceived clarity for one item asking about the frequency of consumption of foods from fast food restaurants. French-speaking participants reported not being sure about whether to include ready-to-heat foods from a grocery store in their answer (e.g. a frozen pizza) whereas English-speaking participants did not find the question unclear.

The 10-item Canadian Food Intake Screener for Adolescents assesses intake over the past week and is multifactorial, capturing key elements of healthy food choices recommendations from the CFG-2019, essentially encouraging intake of fruits, vegetables, protein foods, whole grains and limiting intake of highly processed foods. However, it is not comprehensive in capturing all aspects of a whole diet. Similar to the adult version of the screener [10, 12], the adolescent version does not query all examples of highly processed foods.

This study has several limitations. First, while the sample size was consistent with recommendations for cognitive testing [29–31], there are likely developmental differences among adolescents that influence the accuracy and reliability of the dietary data. Younger adolescents (children aged 10–13 years) may have lower levels of literacy and numeracy compared to older children and therefore may have more difficulty in estimating intakes over the previous week. However, since this study included a greater proportion of 10–13-year-olds than 14–17-year-olds, the screener is likely appropriately tailored for this demographic. Finally, we had more difficulty recruiting racialized French-speaking adolescents and therefore, their perspectives related to dietary intake may have been overlooked.

## Conclusion

The Canadian Food Intake Screener for Adolescents allows for a rapid assessment of food intake relative to the healthy food choices recommendations for children and adolescents within the CFG-2019. Collaboration with a range of advisors, along with assessment of content validity with a panel of experts and cognitive interviews facilitated the development of a simple 10-item screener in both English and French intended for use with children aged 10 to 17 years. Appropriate use of the screener can promote consistent assessment of alignment of children’s dietary intakes with CFG-2019 healthy food choices recommendations in the context of research and population health surveillance. However, further research is needed to develop a scoring system and evaluate the screener’s construct validity and reliability.

## Supplementary Information

Supplementary Material 1.

Supplementary Material 2.

Supplementary Material 3.

## Data Availability

Data are available from the corresponding author upon reasonable request.
