# Supplementary material for: Development of the Canadian food intake screener for adolescents based on Canada’s Food Guide 2019 healthy eating recommendations
Source: Int J Behav Nutr Phys Act. 2025 Oct 21;22:129. doi: 10.1186/s12966-025-01837-1 (PMC12539109; doi:10.1186/s12966-025-01837-1)
Supplement: Supplementary file 1 — Supplementary Material 1. [file 12966_2025_1837_MOESM1_ESM.docx]

**Additional File 1.** Advisors who provided input on the development of the Canadian Eating Practices Screener for Adolescents and the Canadian Food Intake Screener for Adolescents

**French:**

Véronique Gingras, Université de Montréal, Canada

Raphaëlle Jacob, University of Guelph, Canada

Jacynthe Lafrenière, Health Canada

Simone Lemieux, Université Laval, Canada

Isabelle Massarelli, Health Canada

Maude Perreault, Université de Montréal, Canada

Véronique Provencher, Université Laval, Canada

**English:**

Kate Bauer, University of Michigan, USA

Heidi Boyd, Department of Health and Community Services, Government of Newfoundland and Labrador, Canada

Tina Busetto, Department of Health and Social Services, Government of Yukon, Canada

Shawn Brulé, Statistics Canada

Lisa-Anne Elvidge, Health Canada

Katie Loth, University of Minnesota, USA

Alicia E. Martin, University of Guelph, Canada

Isabelle Massarelli, Health Canada

Shannon Olsen, Health Canada

Janis Randall Simpson, University of Guelph, Canada

Joyce Slater, University of Manitoba, Canada

Kathryn Walton, University of Guelph, Canada
