# Supplementary material for: Development of the Canadian food intake screener for adolescents based on Canada’s Food Guide 2019 healthy eating recommendations
Source: Int J Behav Nutr Phys Act. 2025 Oct 21;22:129. doi: 10.1186/s12966-025-01837-1 (PMC12539109; doi:10.1186/s12966-025-01837-1)
Supplement: Supplementary file 2 — Supplementary Material 2. [file 12966_2025_1837_MOESM2_ESM.docx]

**Additional File 2.** Canadian Food Intake Screener for Adolescents

The following questions ask about your eating habits. When answering, please answer based on what you ate during the past week. Consider all meals such as breakfast, lunch, dinner/supper, and snacks.

1. How often did you eat fruit (fresh, frozen, canned, and dried) in the past week? Do not include fruit juices or drinks.

- Never
- 1-2 times per week
- 3-4 times per week
- 5-6 times per week
- 1 time per day
- 2-3 times per day
- 4-5 times per day
- 6 or more times per day

1. How often did you eat vegetables (fresh, frozen, and canned) in the past week? Do not include deep-fried vegetables like French fries, or vegetable juices and drinks.

- Never
- 1-2 times per week
- 3-4 times per week
- 5-6 times per week
- 1 time per day
- 2-3 times per day
- 4-5 times per day
- 6 or more times per day

1. How often did you eat food from fast-food restaurants in the past week? Examples of these foods include:

- hamburgers, hot dogs
- French fries
- poutine
- pizza
- fried chicken and chicken nuggets
- tacos
- sushi
- ice cream, milk shakes
- Never
- 1-2 times per week
- 3-4 times per week
- 5-6 times per week
- 1 time per day
- 2-3 times per day
- 4-5 times per day
- 6 or more times per day

1. How often did you eat processed meats in the past week? Processed meats include:

- hot dogs
- sausages
- deli meats (ham, pastrami/smoked meat, salami)
- beef jerky
- bacon
- Do not include canned fish or canned poultry.
- Never
- 1-2 times per week
- 3-4 times per week
- 5-6 times per week
- 1 time per day
- 2-3 times per day
- 4-5 times per day
- 6 or more times per day

1. How often did you eat protein foods in the past week? Protein foods include:

- eggs
- lean meats and poultry
- nuts and seeds
- fish and shellfish
- milk and dairy products (yogurt, cheese)
- beans, lentils, chickpeas
- fortified soy beverages
- tofu, soybeans, and other soy products

Do not include hot dogs, sausages, deli meats (ham, pastrami/smoked meat, salami), beef jerky, bacon.

- Never
- 1-2 times per week
- 3-4 times per week
- 5-6 times per week
- 1 time per day
- 2-3 times per day
- 4-5 times per day
- 6 or more times per day

1. How often did you eat plant-based protein foods in the past week? Plant-based protein foods include:

- nuts and seeds
- beans, lentils, chickpeas
- fortified soy beverages, tofu, soybeans, and other soy products

Do not include green beans and packaged veggie burgers or other processed plant-based meats.

- Never
- 1-2 times per week
- 3-4 times per week
- 5-6 times per week
- 1 time per day
- 2-3 times per day
- 4-5 times per day
- 6 or more times per day

1. How often did you have sweet drinks in the past week? Sweet drinks include:

- iced tea
- fruit juice
- fruit-flavoured drinks like fruit punch
- regular/diet soda or pop
- sports drinks
- energy drinks
- hot chocolate and chocolate milk
- specialty coffee and teas
- sweetened or artificially flavoured waters
- sweetened plant-based beverages
- Never
- 1-2 times per week
- 3-4 times per week
- 5-6 times per week
- 1 time per day
- 2-3 times per day
- 4-5 times per day
- 6 or more times per day

1. How often did you eat sugary foods in the past week? Sugary foods include:

- cookies
- cakes
- store-bought muffins
- store-bought granola bars
- pastries
- store-bought protein bars
- ice cream
- candy
- chocolate
- sugary breakfast cereals
- Never
- 1-2 times per week
- 3-4 times per week
- 5-6 times per week
- 1 time per day
- 2-3 times per day
- 4-5 times per day
- 6 or more times per day

1. How often did you eat salty snacks in the past week? Salty snacks include:

- chips
- crackers
- pretzels
- popcorn
- Never
- 1-2 times per week
- 3-4 times per week
- 5-6 times per week
- 1 time per day
- 2-3 times per day
- 4-5 times per day
- 6 or more times per day

1. How often did you have whole wheat or whole grain foods in the past week? Whole wheat or whole grain foods include:

- whole wheat or whole grain breads
- whole grain pasta and noodles
- quinoa
- oats
- brown or wild rice
- whole grain breakfast cereals
- Never
- 1-2 times per week
- 3-4 times per week
- 5-6 times per week
- 1 time per day
- 2-3 times per day
- 4-5 times per day
- 6 or more times per day

**Additional File 2.** Questionnaire court canadien sur les apports alimentaires des adolescents

Les questions suivantes portent sur ton alimentation. Pour chaque question, répond en fonction de ce que tu as mangé durant la dernière semaine. Considère tous les repas comme le déjeuner, le dîner, le souper et les collations.

1. Combien de fois as-tu mangé des fruits (frais, congelés, en conserve et secs) durant la dernière semaine? N’inclus pas les jus de fruits ni les boissons aux fruits.

- Jamais
- 1 à 2 fois par semaine
- 3 à 4 fois par semaine
- 5 à 6 fois par semaine
- 1 fois par jour
- 2 à 3 fois par jour
- 4 à 5 fois par jour
- 6 fois ou plus par jour

1. Combien de fois as-tu mangé des légumes (frais, congelés ou en conserve) durant la dernière semaine? N’inclus pas les légumes frits comme les patates frites ni les jus ou cocktails de légumes.

- Jamais
- 1 à 2 fois par semaine
- 3 à 4 fois par semaine
- 5 à 6 fois par semaine
- 1 fois par jour
- 2 à 3 fois par jour
- 4 à 5 fois par jour
- 6 fois ou plus par jour

1. Combien de fois as-tu mangé des aliments qui venaient d’un restaurant de type « fast food » durant la dernière semaine? Voici quelques exemples de ces aliments:

- hamburgers, hot-dogs
- frites
- poutine
- pizza
- poulet frit ou croquettes de poulet
- tacos
- sushi
- crème glacée, lait fouetté
- Jamais
- 1 à 2 fois par semaine
- 3 à 4 fois par semaine
- 5 à 6 fois par semaine
- 1 fois par jour
- 2 à 3 fois par jour
- 4 à 5 fois par jour
- 6 fois ou plus par jour

1. Combien de fois as-tu mangé des viandes transformées durant la dernière semaine? Les viandes transformées incluent:

- hot-dogs
- saucisses
- charcuteries et viandes froides (jambon, pastrami/smoked meat, salami)
- jerky au bœuf
- bacon

N'inclus pas le poisson ou le poulet en conserve.

- Jamais
- 1 à 2 fois par semaine
- 3 à 4 fois par semaine
- 5 à 6 fois par semaine
- 1 fois par jour
- 2 à 3 fois par jour
- 4 à 5 fois par jour
- 6 fois ou plus par jour

1. Combien de fois as-tu mangé des aliments protéinés durant dans la dernière semaine? Les aliments protéinés incluent:

- œufs
- viandes maigres et volaille
- noix et graines
- poissons et crustacées
- lait et produits laitiers (yogourt, fromage)
- fèves/haricots secs, lentilles, pois chiches
- boissons de soya enrichies, tofu ou autres produits de soya

N’inclus pas les hot-dogs, saucisses, charcuteries ou viandes froides (jambon, pastrami/smoked meat, salami), jerky au bœuf, bacon.

- Jamais
- 1 à 2 fois par semaine
- 3 à 4 fois par semaine
- 5 à 6 fois par semaine
- 1 fois par jour
- 2 à 3 fois par jour
- 4 à 5 fois par jour
- 6 fois ou plus par jour

1. Combien de fois as-tu mangé des aliments protéinés d'origine végétales durant la dernière semaine? Les aliments protéinés d'origine végétale incluent:

- noix et graines
- fèves/haricots secs, lentilles, pois chiches
- boissons de soya enrichies, tofu ou autres produits de soya

N’inclus pas les fèves/haricots verts ou jaunes, les hamburgers végétariens et autres viandes transformées d'origine végétale.

- Jamais
- 1 à 2 fois par semaine
- 3 à 4 fois par semaine
- 5 à 6 fois par semaine
- 1 fois par jour
- 2 à 3 fois par jour
- 4 à 5 fois par jour
- 6 fois ou plus par jour

1. Combien de fois as-tu bu des boissons sucrées durant la dernière semaine? Les boissons sucrées incluent :

- thé glacé
- jus de fruits
- boissons aromatisées aux fruits
- boissons gazeuses régulières/diètes
- boissons sportives
- boissons énergisantes
- chocolat chaud et lait au chocolat
- café ou thé contenant du sucre ajouté
- eau aromatisée sucrée
- boissons d’origine végétales sucrées
- Jamais
- 1 à 2 fois par semaine
- 3 à 4 fois par semaine
- 5 à 6 fois par semaine
- 1 fois par jour
- 2 à 3 fois par jour
- 4 à 5 fois par jour
- 6 fois ou plus par jour

1. Combien de fois as-tu mangé des aliments sucrés durant la dernière semaine? Les aliments sucrés incluent:

- biscuits
- gâteaux
- muffins achetés en magasin
- barres granola ou barres tendres achetées en magasin
- pâtisseries
- barres protéinées achetées en magasin
- crème glacée
- bonbons
- chocolat
- céréales à déjeuner sucrées
- Jamais
- 1 à 2 fois par semaine
- 3 à 4 fois par semaine
- 5 à 6 fois par semaine
- 1 fois par jour
- 2 à 3 fois par jour
- 4 à 5 fois par jour
- 6 fois ou plus par jour

1. Combien de fois as-tu mangé des grignotines salées durant la dernière semaine? Les grignotines salées incluent :

- chips
- craquelins
- pretzels
- popcorn
- Jamais
- 1 à 2 fois par semaine
- 3 à 4 fois par semaine
- 5 à 6 fois par semaine
- 1 fois par jour
- 2 à 3 fois par jour
- 4 à 5 fois par jour
- 6 fois ou plus par jour

1. Combien de fois as-tu mangé des aliments de blé entier ou à grains entiers durant la dernière semaine? Les aliments de blé entier ou à grains entiers incluent :

- pains de blé entier ou à grains entiers
- pâtes et nouilles de blé entier ou à grains entiers
- quinoa
- avoine
- riz brun ou riz sauvage
- céréales à déjeuner de blé entier ou à grains entiers
- Jamais
- 1 à 2 fois par semaine
- 3 à 4 fois par semaine
- 5 à 6 fois par semaine
- 1 fois par jour
- 2 à 3 fois par jour
- 4 à 5 fois par jour
- 6 fois ou plus par jour
